# Impact of Anesthetics on Immune Functions in a Rat Model of Vagus Nerve Stimulation

**DOI:** 10.1371/journal.pone.0067086

**Published:** 2013-06-26

**Authors:** Chloé A. Picq, Didier Clarençon, Valérie E. Sinniger, Bruno L. Bonaz, Jean-François S. Mayol

**Affiliations:** 1 Institut de Recherche Biomédicale des Armées, Antenne de La Tronche, Centre de Recherche du Service de Santé des Armées, La Tronche, France; 2 Stress et Interactions Neuro-Digestives Grenoble Institut des Neurosciences, Centre de Recherche INSERM 836 UJF-CEA-CHU, La Tronche, France; 3 Clinique Universitaire d’Hépato-Gastroentérologie, CHU de Grenoble, Grenoble, France; Hannover Medical University (MHH), Germany

## Abstract

Vagus nerve stimulation (VNS) has been successfully performed in animals for the treatment of different experimental models of inflammation. The anti-inflammatory effect of VNS involves the release of acetylcholine by vagus nerve efferent fibers inhibiting pro-inflammatory cytokines (e.g. TNF-α) produced by macrophages. Moreover, it has recently been demonstrated that splenic lymphocytic populations may also be involved. As anesthetics can modulate the inflammatory response, the current study evaluated the effect of two different anesthetics, isoflurane and pentobarbital, on splenic cellular and molecular parameters in a VNS rat model. Spleens were collected for the characterization of lymphocytes sub-populations by flow cytometry and quantification of cytokines secretion after *in vitro* activation. Different results were observed depending on the anesthetic used. The use of isoflurane displayed a non-specific effect of VNS characterized by a decrease of most splenic lymphocytes sub-populations studied, and also led to a significantly lower TNF-α secretion by splenocytes. However, the use of pentobarbital brought to light immune modifications in non-stimulated animals that were not observed with isoflurane, and also revealed a specific effect of VNS, notably at the level of T lymphocytes’ activation. These differences between the two anesthetics could be related to the anti-inflammatory properties of isoflurane. In conclusion, pentobarbital is more adapted than isoflurane in the study of the anti-inflammatory effect of VNS on an anesthetized rat model in that it allows more accurate monitoring of subtle immunomodulatory processes.

## Introduction

The vagus nerve plays an important role in the control of inflammation and is the principal component of the “reflex control arc of immunity” [1]. This arc is composed of sensory afferent fibers of the vagus nerve which detect the presence of molecules secreted when inflammation or infection occurs at the periphery. These afferent fibers convey the information to the central nervous system (CNS). In response, the efferent fibers of the vagus nerve are activated to transmit the information and modulate the peripheral immune response. Therefore, the vagus nerve has a double action: informing the CNS of a peripheral inflammation via its afferents, and modulating inflammation via its efferent fibers [2], [3].

Low-frequency vagus nerve stimulation (VNS) has been successful for the treatment of different animal models of inflammation such as septic shock, pancreatitis, ischaemia-reperfusion, postoperative ileus, and arthritis (see Tracey for review [1]). We have previously demonstrated a potential role for VNS as a therapeutic strategy for the treatment of chronic inflammatory bowel disease in a rat model of colitis [4]. The anti-inflammatory effect of VNS is mediated by the activation of the vagus nerve efferent fibers, which in turn activate the cholinergic anti-inflammatory pathway. This pathway, discovered by Tracey’s group [5], is complementary to the hypothalamic-pituitary-adrenal axis, classically activated by vagus nerve afferents. Vagus nerve efferent fibers release acetylcholine, which binds to macrophages nicotinic receptors. This binding inhibits the translocation of the transcription factor NF-κB into the nucleus which leads to the inhibition of the secretion of pro-inflammatory cytokines such as TNF-α.

However, other pathways implicating different immune cells could also be activated by VNS. Recent studies have shown that the spleen has a crucial role in the anti-inflammatory effect of the vagus nerve [6], [7], [8]. In order to study the implication of the spleen in the anti-inflammatory effect of VNS in the gut, we decided to develop an anesthetized rat model. Indeed, the interest of working on an anesthetized rat model was to study early molecular and cellular mechanisms activated in response to VNS and inflammation, which is not possible when working on an implanted and freely moving rat model. Nevertheless, as anesthetics may cause interferences especially at the immunomodulatory level, the evaluation of pitfalls inherent to the use of anesthetics seemed important. The anti-inflammatory effect of VNS might be over- or under-estimated depending on the anesthetic used.

Isoflurane is commonly used in studies where long-term anesthesia is necessary because of different positive aspects: it is easy to use, it causes low levels of stress for the induction of anesthesia, its quality is readily monitored, and the dosage can be easily modified according to the animal’s condition during the surgical intervention. However, isoflurane can potentially interfere at different levels, such as the central nervous system [9], [10] or the immune system. Indeed, isoflurane has anti-inflammatory properties which can be sources of interferences in studies concerning inflammation [11], [12], [13], [14], [15], [16], [17], [18]. Moreover, the use of isoflurane has not yet been studied in a VNS rat model.

On the other hand, pentobarbital is not known to have immunoregulatory properties and is often used as control anesthetic in studies concerning the effects of anesthetics on inflammation [19], [20], [21], [22]. However, pentobarbital is more commonly used in short-term anesthesia because of its cardio-pulmonary depressor properties and the difficulty of assessing anesthesia depth. As VNS induces a small decrease in heart rate in rats [23], [24], [25] and displays immunoregulatory properties, we investigated the impact of two different anesthetics: isoflurane and pentobarbital, to minimize the adverse effects of anesthesia in our experimental model of VNS. For this purpose, our study focused on molecular and cellular mechanisms occurring in the spleen in response to the different anesthetics and VNS protocols.

## Materials and Methods

### Ethics Statement

All animals were treated according to guidelines approved by the European legislation for the care of laboratory animals. Protocols (ID#: 2009/21.0) were approved specifically for this study by the IRBA Animal Care and Use Committee (IACUC).

### Animals

Adult male Sprague-Dawley rats (260–280 g) (Janvier, Le Genest St Isle, France) were housed in a controlled environment (12 h light/dark cycles, 20–22°C) with food and water *ad libitum*. Rats were allowed a minimum of 7 days to adapt to housing conditions before any manipulation.

### Anesthesia

Two experimental protocols of anesthesia were used: volatile anesthesia with isoflurane (Abott, Rungis, France) and intraperitoneal injection of pentobarbital (Ceva Santé Animale, Libourne, France).

Five experimental groups were used for each anesthesia protocol, with 10 rats in each: (1) control group without surgery; (2) VNS 3 hours: anesthetized, operated, and stimulated for 3 hours; (3) VNS 20 minutes: anesthetized, operated, and stimulated for 20 minutes; (4) sham 3 hours: anesthetized, operated, and not stimulated; (5) sham 20 minutes: anesthetized, operated, and not stimulated.

Every vagus nerve stimulation or sham period was preceded and followed by 30 minutes of resting time under anesthesia. The total time of anesthesia was respectively 1 hour and 20 minutes for the 20 minutes stimulated groups and 4 hours for the 3 hours stimulated groups.

Pentobarbital (intraperitoneale injection, 125 µl/100 g) anesthetized rats were placed on electric heating pads and allowed to breathe room air spontaneously. Isoflurane anesthetized rats were placed in an airtight chamber where 3.5% isoflurane in 3l/min oxygen was delivered to initiate anesthesia. Rats were then placed on electric heating pads and anesthesia was maintained at 2.5% isoflurane in 1l/min oxygen. Body temperature was monitored with a rectal probe and maintained at 37°C. Oxygen saturation and heart rate were monitored during the total length of anesthesia (including surgery).

Experiments were performed simultaneously on 3 rats per day (one animal from the control group; one from the non stimulated group, and one from the stimulated group).

### Surgical Procedures and Vagus Nerve Stimulation Parameters

VNS was performed as previously described [4]. An incision was made over the ventral region of the neck. The left vagus nerve and the carotid artery were individualized and the vagus nerve was separated from the carotid artery. A bipolar electrode (Harvard Apparatus, France) was gently placed on the vagus nerve and connected to a stimulation chain: S88 stimulator, SIU5 isolation unit, CCU1 constant current unit (Grass Technologies, Astro-Med, RI, US). VNS was performed using biphasic pulses with the following parameters: 0.5 mA, 5 Hz, 500 µs pulse width and cycle of 30 seconds ON and 300 seconds OFF.

At the end of the experiment, rats were euthanized with an intracardiac injection of pentobarbital (Dolethal®, 200 mg/kg). A laparotomy was performed and the spleen was collected.

### Spleen Preparation for flow Cytometry Analysis

The spleen was placed in 10 ml of cold phosphate buffer saline (PBS) supplemented with bovine serum albumin (BSA) 1%. The spleen was placed on a strainer (100 µm) and ground to mechanically dissociate the cells and obtain single-cell suspension. Isolated cells were then diluted with 20 ml of hypotonic lysis buffer (ammonium chloride), allowing the lysis of red blood cells. After centrifugation, cells were washed with PBS and filtered on a 40 µm strainer. After a last wash with PBS, the cells were resuspended in 10 ml of PBS-BSA 1% and counted using Trypan blue in Malassez cell in order to determine the total number of cells derived from one whole spleen.

Splenocytes were stained with fluorescent antibodies in order to characterize the different lymphocyte sub-populations present in the spleen. Each staining was performed on one million cells. Cells were incubated for 45 min with a mix of antibodies and PBS BSA 1% at 4°C. For each staining, an isotypic control was done to determine the positive threshold of antigens according to the strategy of “Fluorescence Minus One–FMO” [26]. After incubation, cells were washed with PBS, centrifuged, and the supernatants were removed. Pelleted cells were resuspended with PBS and Hoechst 33258 (Sigma, L’Isle D’Abeau, France) for viability assessment.

The following antibodies coupled with fluorochromes were used:

APC Mouse Anti-Rat CD3, FITC Mouse Anti-Rat CD8a, PE-Cy5 Mouse Anti-Rat CD4, PE Mouse Anti-Rat CD25, PE Mouse Anti-Rat CD45RA, FITC Mouse Anti-Rat CD161a, PE Mouse Anti-Rat γδ T-Cell Receptor, PerCP Mouse Anti-Rat αβ T-Cell Receptor (BD Biosciences, Le Pont de Claix, France).

The analyses were performed on a FACS LSRII (BD Biosciences). FACS data were interpreted using Flowjo software (Tree Star, Ashland, USA). On the basis of the percentage measured and the total cell numeration, an absolute number of each sub-population per organ was calculated.

### Quantification of Cytokines

One million unstained splenocytes from the single-cell suspension were placed in cell culture plates (96-well) coated with anti-CD3 antibody (Abd Serotec, Oxford, UK) for 48 hours. Wells were then washed with PBS and filled with one million cells with soluble anti-CD28 (Abd Serotec, Oxford, UK), RPMI and foetal calf serum (FCS) 10%. Plates were placed at 37°C, 5% CO_2_ for 48 hours. Supernatants were collected and stored at −80°C before cytokine quantification. To quantify cytokine secretion, Cytometric Beads Array (CBA) Rat Soluble Protein Flex Sets (BD Biosciences) were used according to the manufacturer’s instructions. The quantified cytokines were the following: IL-4, TNF-α, IL-1α, IFN-γ, IL-10 and IL-6.

### Statistical Analysis

Statistical analyses were performed on GraphPad Prism software version 5.00 (GraphPad Software, San Diego, USA). Data were analyzed with the non-parametric test Mann-Whitney to evaluate differences between two groups. A p value of less than 0.05 was considered as statistically significant.

## Results

The functionality of the vagus nerve was monitored by recording the electrocardiogram of each animal, during the whole experiment. A non-significant (mean ± SEM 6±5%) decrease of the heart rate was observed during the ON VNS period; the heart rate went back to normal during the OFF period. This effect was observed even after 3 hours of VNS, demonstrating that the vagus nerve was not altered after long term VNS (data not shown).

Splenic lymphocyte sub-populations isolated from anesthetized rats with isoflurane or pentobarbital were analyzed by flow cytometry to evaluate the impact of both anesthetics on our experimental VNS model. Different results were observed depending on the anesthetic used. Firstly, we compared the effect of the two anesthetics on sham groups in order to highlight their effect on cellular parameters at basal level. Secondly, we evaluated how these anesthetics may interfere with the anti-inflammatory effect of VNS.

### 1- Effect of Pentobarbital and Isoflurane on Sham Operated Animals

**Figure 1:**
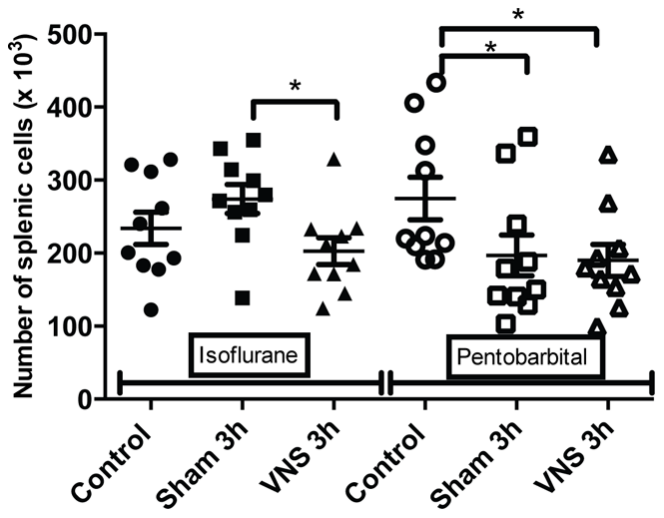
Quantification of splenic T CD3 for isoflurane and pentobarbital anesthetized rats. The number of splenic T CD3 lymphocytes isolated from rats anesthetized with isoflurane or pentobarbital was analysed with flow cytometry in the different groups (control, sham 3 h, VNS 3 h). n = 10 rats per groups. *: p<0.05. A significant decrease was observed for the VNS 3 h group compared to the sham 3 h group for the isoflurane anesthetized rats. A significant decrease of the number of splenic T CD3 lymphocytes was observed for the operated groups (sham 3 h, VNS 3 h) compared to the control group for the pentobarbital anesthetized rats (p<0.05).

**Figure 2:**
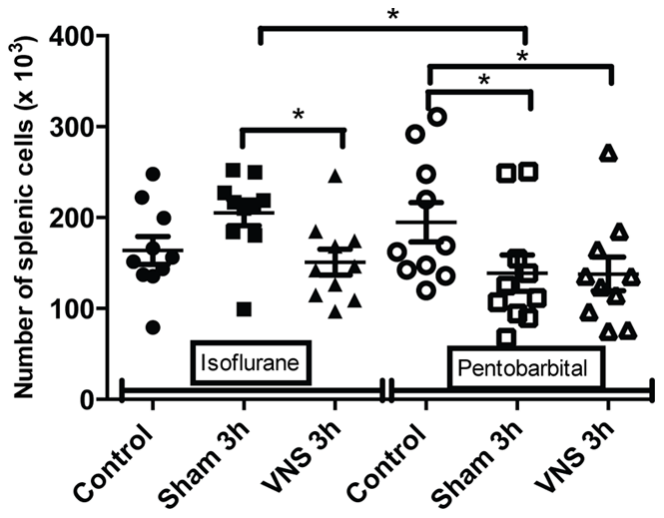
Quantification of splenic T CD4 for isoflurane and pentobarbital anesthetized rats. The number of splenic T CD4 lymphocytes isolated from rats anesthetized with isoflurane or pentobarbital was analysed with flow cytometry in the different groups (control, sham 3 h, VNS 3 h). n = 10 rats per groups. *: p<0.05. A significant decrease was observed for the VNS 3 h group compared to the sham 3 h group for the isoflurane anesthetized rats. A significant decrease was also observed for the operated groups (sham 3 h, VNS 3 h) compared to the control group for the pentobarbital anesthetized rats. Moreover, a significant decrease was observed between both sham 3 h groups (pentobarbital versus isoflurane).

**Figure 3:**
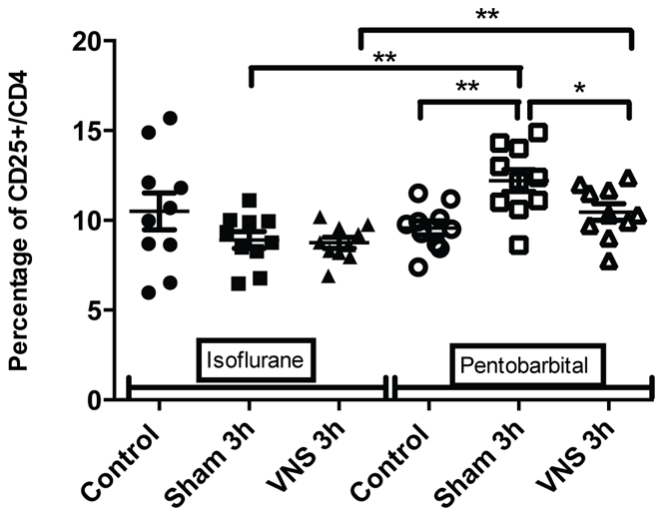
Splenic T CD4 activation for isoflurane and pentobarbital anesthetized rats. The splenic T CD4 activation (percentage of CD25+/CD4 LT) was analysed with flow cytometry for the different conditions (control, sham 3 h, VNS 3 h) with either isoflurane or pentobarbital anesthesia. n = 10 rats per groups. *: p<0.05, **: p<0.01. Modulations of T CD4 activation were observed with pentobarbital but not with isoflurane. A significant increase was observed for the sham 3 h group compared to the control group, as well as a significant decrease for the VNS 3 h group compared to the sham 3 h group. Moreover, a significant increase was observed for both VNS and sham 3 h groups from the pentobarbital anesthetized animals compared to isoflurane anesthetized groups.

**Figure 4:**
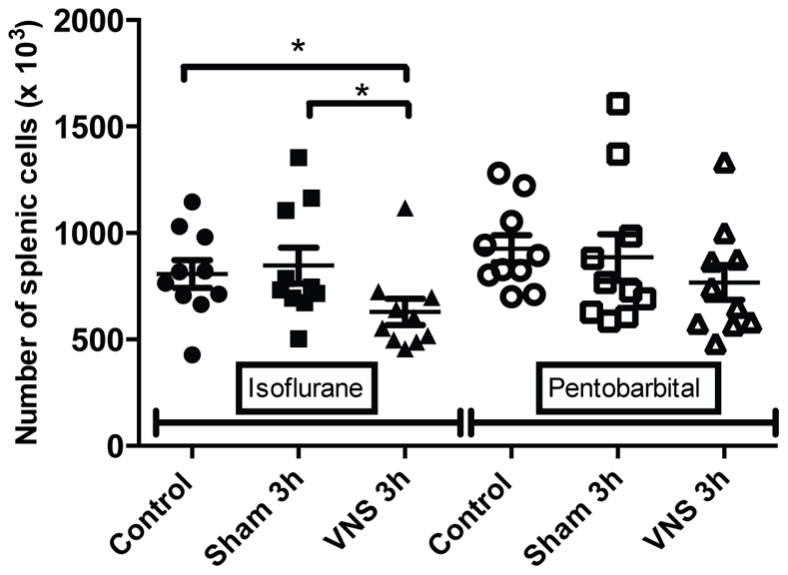
Modulation of total splenic cellularity for isoflurane and pentobarbital anesthetized rats. The total splenic cellularity from rats anesthetized with isoflurane or pentobarbital was determined using counting with Trypan blue in Malassez cell for the different groups (control, sham 3 h, VNS 3 h). n = 10 rats per groups. *: p<0.05. A significant decrease was observed for the VNS 3 h group compared to the sham 3 h and control groups (p<0.05) for the isoflurane anesthetized rats. In contrast, no significant effect was observed for the pentobarbital anesthetized rats.

**Figure 5:**
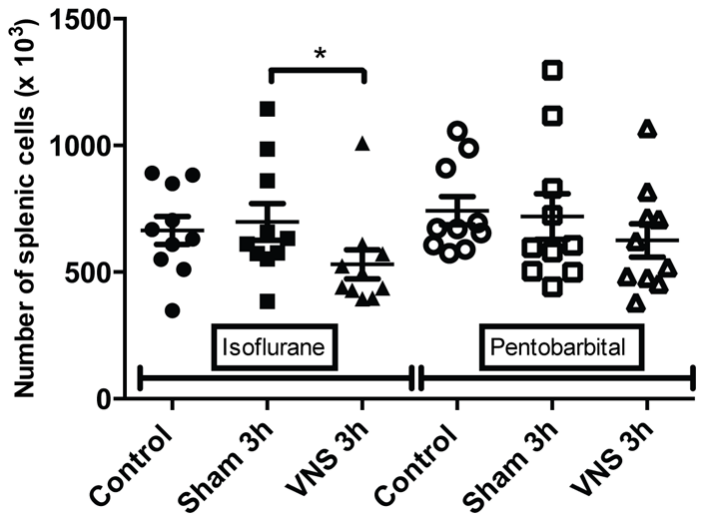
Quantification of total lymphocytes for isoflurane and pentobarbital anesthetized rats. The number of total splenic lymphocytes isolated from rats anesthetized with isoflurane or pentobarbital was analysed with flow cytometry in the different groups (control, sham 3 h, VNS 3 h). n = 10 rats per groups. *: p<0.05. A significant decrease was observed for the VNS 3 h group compared to the sham 3 h group for the isoflurane anesthetized rats. No significant effect was observed for the pentobarbital anesthetized rats.

**Figure 6:**
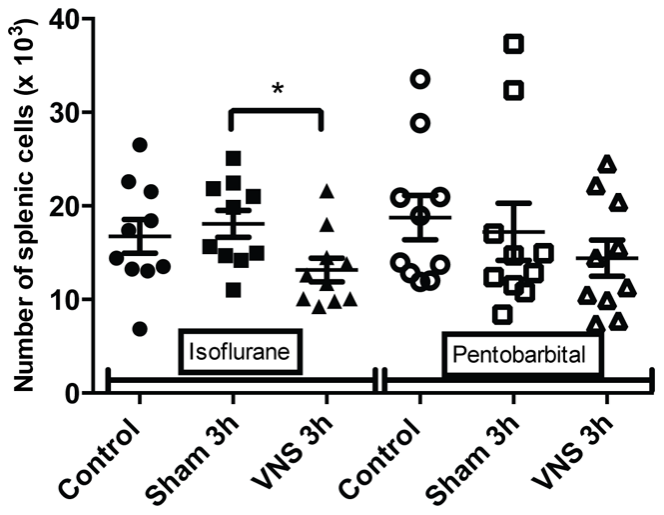
Quantification of splenic T CD4 CD25 lymphocytes for isoflurane and pentobarbital anesthetized rats. The number of splenic T CD4 CD25 lymphocytes isolated from rats anesthetized with isoflurane or pentobarbital was analysed with flow cytometry in the different groups (control, sham 3 h, VNS 3 h). n = 10 rats per groups. *: p<0.05. A significant decrease was observed for the VNS 3 h group compared to the sham 3 h group for the isoflurane anesthetized rats. No significant effect was observed for the pentobarbital anesthetized rats.

Flow cytometry results on splenocytes isolated from pentobarbital anesthetized rats showed a significant decrease of the number of T lymphocytes (−27%, p<0.05) (figure 1) and CD4 T lymphocytes (−29%, p<0.05) (figure 2) for all the operated groups (sham 3 h and VNS 3 h groups) compared to the control group (not operated). These variations in the number of T cells could reflect an early inflammatory response induced by the surgery used to place the stimulation electrode. A significant increase (+27%, p<0.01) in lymphocytes activation was also observed for the sham 3 h group compared to the control group (figure 3). However, on rats anesthetized with isoflurane, there was no significant effect on splenocytes from the sham 3 h group compared to the control group for total cellularity (figure 4) and for every sub-population studied (figures 1,2,3,5,6). Moreover, when comparing the effect of both anesthetics (isoflurane versus pentobarbital) on sham 3 h groups, a significant difference was observed for the T CD4 lymphocytes sub-population (figure 2) and for lymphocytes activation (figure 3). No difference was observed between the two anesthetics on sham 20 min groups (data not shown).

### 2-Interactions between the Type of Anesthetics and VNS

Data of rats anesthetized with isoflurane showed a modulation in the number of cells. A significant decrease (−25%, p<0.05) of total spleen cellularity was observed for the VNS 3 h group compared to the sham 3 h and control group (figure 4). This significant decrease for the VNS 3 h group was also found for other cellular sub-types such as total lymphocytes (−24%, p<0.05) (figure 5), T lymphocytes (−26%, p<0.05) (figure 1), T CD4 lymphocytes (−26%, p<0.05) (figure 2), activated T CD4 lymphocytes (−27%, p<0.05) (CD25+) (figure 6). Moreover, a non significant decrease was observed for the VNS 3 h group compared to the sham 3 h group for T CD8 lymphocytes, B lymphocytes and NK cells (data not shown).

Different results were obtained from splenocytes isolated from rats anesthetized with pentobarbital. No decrease of the total splenic cellularity was observed for splenocytes of the pentobarbital treated rats, unlike those of the isoflurane anesthetized rats (figure 4). However, a decrease in lymphocytes activation (−14%, p<0.05) was observed for the 3 h VNS group compared to the sham group (figure 3). The anti-inflammatory effect of VNS could inhibit CD4 lymphocytes activation. Moreover, a significant difference was observed between both VNS 3 h groups (isoflurane versus pentobarbital) concerning lymphocytes activation (figure 3). However, no effect was seen for the 20 min VNS and sham groups (data not shown).

### 3-Cytokines Secreted by Cultured Splenocytes

**Figure 7:**
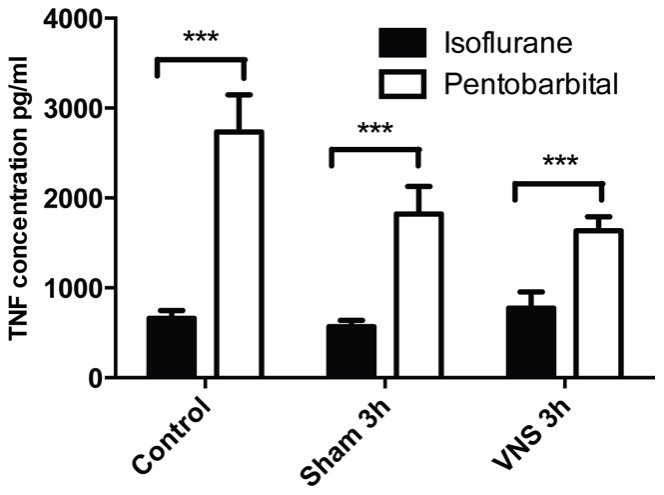
Quantification of tumor necrosis factor-α secreted by cultured splenocytes for isoflurane and pentobarbital anesthetized rats. The concentration of tumor necrosis factor-α secreted by cultured splenocytes isolated from isoflurane or pentobarbital anesthetized rats was determined using the cytometric bead array technique. ***: p<0.001. Different levels of TNF-α secretion were observed depending on the anesthetic used. Regardless of the conditions (control, sham 3 h or VNS 3 h) the level of TNF-α secretion was significantly lower for the cultured splenocytes isolated from isoflurane anesthetized groups compared to pentobarbital anesthetized group.

A quantification of secreted cytokines was carried out in the supernatants of cultured splenocytes. IL-6 and IL-1α were considered as not detectable. IL-4, IL-10 and IFN-γ were detectable but no significant difference was observed between activated splenocytes derived from isoflurane and pentobarbital anesthetized animals (data not shown). However, important level differences were observed for the secretion of the pro-inflammatory cytokine TNF-α. The secretion level of TNF-α was significantly lower (2 to 3 times, p<0.001) for all the experimental groups anesthetized with isoflurane compared to the groups anesthetized with pentobarbital (figure 7). The use of isoflurane inhibits TNF-α secretion by cultured and activated splenocytes.

## Discussion

In this study we evaluated the impact of two different anesthetics on splenic cellular and molecular parameters to minimize their potential adverse effects in our VNS model. We have previously shown that low-frequency VNS (5 Hz), chronically performed in non-anesthetized animals, has anti-inflammatory properties in a model of experimental colitis in rats [4]. Different pathways seem to be implicated in this anti-inflammatory effect: the cholinergic anti-inflammatory pathway involving macrophages, described in 2000 by Tracey’s group [5], and an additional pathway implicating other immune cells. Recent data have shown a crucial role of the spleen in the peripheral anti-inflammatory effect of VNS [7]. In order to study the involvement of the spleen in the anti-inflammatory effect of VNS, we decided to develop an anesthetized rat model. However, given that anesthetics may cause bias, it seemed important to previously evaluate the pitfalls inherent to the use of an anesthetic for an immune-modulation model. Different studies have shown that isoflurane may have anti-inflammatory properties in different models [19], [22], [27], [28]; whereas pentobarbital has been shown to have cardio-pulmonary depressor properties but no anti-inflammatory effect has been detected until now. To be sure to use the most relevant anesthetic for our experimental VNS model, we performed a comparative study of isoflurane and pentobarbital on our model. For this purpose, rats were either anesthetized with an inhalation of isoflurane or with an intraperitoneal injection of pentobarbital.

For the VNS and sham 20 min groups anesthetized for a total time of 1 h20 min no significant results were observed. The immunomodulatory effect of isoflurane might be observable only after a greater delay between anesthesia and tissue collection. Hofstetter et al. have demonstrated that a 50 seconds exposure of isoflurane as a pretreatment before 4 hours of pentobarbital-fentanyl anesthesia in an endotoxemic rat model allowed a significant decrease of plasma levels of TNF-α and IL-1β [22]. We hypothesized that immunomodulatory effects of isoflurane may not be observable shortly after the beginning of anesthesia but only after the onset of non-immediate molecular and cellular mechanisms.

For the VNS and sham 3 h groups anesthetized for 4 hours, three different effects were observed. Firstly, concerning splenic cellularity, the use of isoflurane brought to light a significant decrease for the VNS group compared to the sham group for total lymphocytes and all the other lymphocytes sub-populations. These results showed that VNS did not have a significant effect on a particular subtype of lymphocyte population but had a global effect on all the different sub-populations. Even if this VNS effect appears as non specific, it draws out an effect of VNS on cell trafficking that may modulate a potential immune response. This was not observed with pentobarbital which revealed other modulations not observable with isoflurane. Indeed, a significant decrease of the number of T lymphocytes was observed for all the operated groups compared to the control group (non-operated). This result was also observed for the number of T CD4 lymphocytes (significant for the 3 h groups and a trend for the 20 min groups). It was not the case for the other subpopulations. This decrease of T lymphocytes for the different operated groups compared to the control group could be the result of a small inflammation caused by the surgery performed to place the stimulation electrode on the vagus nerve. T lymphocytes could leave the spleen to migrate to the site of inflammation. Consequently, the use of pentobarbital allows us to highlight precise variations of cellularity in the spleen and to monitor dynamic cellular processes.

Secondly, significant modulations of T CD4 lymphocytes’ activation were observed with the use of pentobarbital but not with isoflurane. A significant increase of the percentage of CD25+ cells in the CD4+ lymphocyte population was observed for the sham 3 h group compared to the control group. This increase could also be explained by a slight inflammation and a potential cellular response in lymphoid organs due to the effect of the surgical procedure to implant the stimulation electrode. Moreover, a significant decrease of T CD4 activation for the VNS 3 h group compared to the sham 3 h group was observed. VNS potentially has an action on lymphocytes’ activation and expression of receptors involved in the inflammatory response (CD25 = α chain of IL-2 receptor). In our model, variations of lymphocytes’ activation showing VNS anti-inflammatory effect were only seen with the use of pentobarbital.

Finally, a significant effect was observed at the level of TNF-α secretion by cultured and CD3/CD28 activated splenic lymphocytes. Even though the secretion levels of IFN-γ and IL-10 were comparable according to the anesthetic used, TNF-α secretion was significantly lower for the isoflurane anesthetised groups compared to pentobarbital anesthetised groups. This difference shows that the anesthetics have no effect on lymphocytes’ activation *in vitro*; however, the use of isoflurane significantly impacts the secretion level of a specific cytokine, TNF-α. We hypothesize that isoflurane inhibits TNF-α secretion via the NF-κB pathway as described previously [13]. Other pathways may not be inhibited like the c-jun/AP-1 pathway implicated in the IFN-γ secretion [29], [30].

On a rat model without inflammation, pentobarbital anesthesia produced specific modulations at the cellular dynamic level, lymphocytes’ activation, and TNF-α secretion unlike isoflurane anesthesia, which caused a global modulation on every lymphocyte sub-population studied. These different processes are crucial for the understanding of VNS effect that could be hidden by the use of isoflurane. Significant differences were observed between both sham 3 h groups (isoflurane versus pentobarbital) for the number of T CD4 lymphocytes and lymphocytes’ activation. This was also the case for both VNS 3 h groups (isoflurane versus pentobarbital) at the level of lymphocytes’ activation. Our data confirms the different effects that the two anesthetics have on our experimental model. These differences could be explained by the anti-inflammatory properties [19], [22], [27], [28] of isoflurane, whereas pentobarbital is not known to act on the inflammation process, and is commonly used as a control anesthetic in studies on anesthetic’s effects on inflammation [19], [20], [22], [31]. Moreover, isoflurane inhibits the NF-κB pathway which plays a key role in the anti-inflammatory effect of VNS [18], [32].

Although the use of isoflurane is more appropriate for long-term anesthesia, it is not the suitable anesthetic to study the anti-inflammatory effect of VNS in our model. Even if pentobarbital is more complex to use, it appears to be more appropriate to evaluate precise cellular responses implicated in the immunomodulation process of VNS. In conclusion, on the basis of these results, the choice of anesthetic must be carefully adapted to the experimental model, particularly in animal models of immunoinflammatory disorders.
